# The Different Patterns of Over-the-Counter Nonsteroidal Anti-Inflammatory Drugs or Analgesics Use in Patients with Chronic Kidney Disease and the General Population

**DOI:** 10.3390/healthcare10102035

**Published:** 2022-10-14

**Authors:** Maria Mulka-Gierek, Natalia Krata, Bartosz Foroncewicz, Leszek Pączek, Krzysztof Mucha

**Affiliations:** 1 Department of Immunology, Transplantology and Internal Diseases, Medical University of Warsaw, 02-006 Warsaw, Poland; 2 Institute of Biochemistry and Biophysics, Polish Academy of Sciences, 02-106 Warsaw, Poland

**Keywords:** chronic kidney disease, general practice, NSAID, OTC, pain killer, side effect, survey

## Abstract

Nonsteroidal anti-inflammatory drugs (NSAIDs) and analgesics are the most commonly used drugs worldwide and their availability over-the-counter is increasing. The aim of this study was to examine the frequency of their use as well as the awareness of the associated risk of side effects in patients with chronic kidney disease (CKD) compared to the patients at general practice (GP) offices. We found that 88.5% of the CKD and 97.1% of the GP group used NSAIDs and/or analgesics (*p* < 0.0001). Paracetamol was chosen the most often by both study groups, but the proportion of patients taking paracetamol was significantly higher in the CKD group (*p* < 0.006). On the contrary, the proportion of patients taking ibuprofen was significantly higher in GP group (*p* < 0.0001). Furthermore, almost 37% of CKD and 60% of GP patients never consult with their doctor before taking NSAIDs or analgesics. The influence of advertisements on the decision to take these drugs was found to be marginal in both groups. In conclusion, the NSAIDs and/or analgesics use is very common. The differences between the studied cohorts in self-decision making and the type of drugs used between the studied cohorts warrant tailored educational approaches.

## 1. Introduction

Nonsteroidal anti-inflammatory drugs (NSAIDs) and analgesics are the most commonly used drugs worldwide and their availability over-the-counter (OTC) is increasing. For example, in the United States, the sales of these drugs are growing yearly, reaching the annual value of approximately 16 billion USD in 2019. Moreover, its further dynamic growth is estimated to 24.7 billion USD in 2027 [1]. Their availability and excessive advertisement may give the illusion that they can be taken without repercussions. This is a worrying phenomenon as most people consider OTC drugs safe [2,3]. We previously reported that the frequency of NSAIDs and analgesics self-use is very high even in a such high risk patient populations such as kidney and liver transplant recipients [4,5]. However, using them against recommendations or unintentionally taking the same substances sold under different trade names may cause serious health consequences. The NSAIDs and analgesics might have the detrimental impact on the function of the heart, central nervous system, gastrointestinal (GI) tract, kidneys and liver [6]. Furthermore, they may cause tachyphylaxis, which would require patients to take higher or more frequent doses to achieve expected relief, leading to addiction [7].

The most common and well-described complication of NSAIDs usage is duodenal and gastric mucosal lesions, reported in at least one third of patients [6,8]. However, NSAIDs also impact lower GI tract causing enteropathy manifested as, for example, iron deficiency anemia, indigestion or abdominal pain [9]. The enteric lesions such as erosions or loss of villi or ulcers can be detected in two thirds of chronic NSAIDs users [10]. Large number of reports have associated NSAIDs with cardiovascular diseases [11,12,13]. Early studies indicated an association between NSAIDs use and increased risks of heart failure or elevated blood pressure. Only the subsequent studies found a link between NSAIDs use and an increased risk of thrombotic events, even after a usage period of less than one week [14]. Accordingly, NSAIDs users have an increased risk of intracerebral bleeding and stroke, especially those who have a history of cerebral ischemic events [15]. These drugs might also affect the function of the central nervous system, causing e.g., memory or sleep disturbances, confusion, cognitive impairment and even hallucinations [7,16,17]. In addition to these, NSAIDs use may induce asymptomatic side effects such as hepato- and/or nephrotoxicity [6]. The latter, added to other side effects, seem particularly dangerous for patients with preexisting chronic kidney diseases (CKD). An altered drug metabolism and its reduced excretion, superimposed onto frequent comorbidities, associated with CKD, increase susceptibility to the adverse drug effects of NSAIDs in these patients. Their use has been associated with acute kidney injury, progressive loss of glomerular filtration rate (GFR), electrolyte disturbances, and hypervolemia with the exacerbation of heart failure and hypertension. The clinical presentation of nephrotoxicity syndromes depends on the GFR. Of note, the pain burden among CKD patients is high whereas safety data concerning NSAIDs use are limited in this subgroup of patients [18].

The aim of this study was to examine the frequency of NSAIDs and analgesics use, the reasons for use, the awareness of the side effects associated with their usage and factors influencing the drug choice in CKD patients compared to patients at general practice (GP) offices.

We believe that the innovative proposals of this project may improve the understanding of NSAIDs and analgesics risk factors among CKD patients, the general population, who is also susceptible for OTC abuse, and healthcare personnel. The advantage of this project might be the discovery of new directions for NSAIDs prescription. We are aware of disadvantages such as the questionnaire’s design, that may be unintelligible especially for elderly patients, which may influence the study outcomes.

## 2. Materials and Methods

### 2.1. Study Design

This was a descriptive survey study that included 226 CKD patients from nephrology out-patient clinic of Medical University of Warsaw (single center) and 345 patients from a GP office from single medical center in Southern Poland. Patients were randomly selected for enrollment and surveyed.

A quantitative design based on two surveys was chosen to meet the study aim: 31 closed questions for CKD and 22 closed questions for GP patients were designed (Appendix A and Appendix B). To enable efficient product recognition, the surveys were accompanied with the album of pictures presenting packages of NSAIDs and analgesics available on the market. The list of these products is summarized in a table presented in Appendix C. The surveys were performed at different time points. The CKD group was surveyed first, and the GP questionnaire was modified based on the CKD answers. Finally, 12 questions were identical for both studied groups and used for comparisons (Appendix D).

Study participation was voluntary and anonymous. The local ethics committee approved the study design, which did not require obtaining written informed consent from the participants.

Patients were surveyed during their routine visits. The survey was administered and the data was collected face-to-face by a registered nurse. To reduce the likelihood of hypothesis guessing, the interviewees were blinded to the research hypotheses. 

### 2.2. Statistical Analysis

The data were analyzed with Statistica 13.3 (StatSoft) and GraphPad 9.3 (Prism) software. A Chi-square (Chi^2^) Test and Fisher’s exact Test were performed for evaluation of statistical difference between selected variants of answers and groups. The Kruskal-Wallis Test was used to compare the differences in creatinine level in previous CKD patient’s history. The level of statistical significance was set as *p* < 0.05. 

## 3. Results

### 3.1. Patient’s Characteristics

#### 3.1.1. CKD Cohort

The CKD patients were aged from 19 to 91 years old (median 60.5 years), 62.4% were females and 37.6% males. Most of them (64.6%) were in retirement, pension age or unemployment. The mean (SD) of the estimated glomerular filtration rate (eGFR) was 58.16 (27.9) at the time of survey and it had not changed significantly within previous 5 years. The most common etiologies of CKD were IgA nephropathy, diabetic, polycystic kidney diseases and focal segmental glomerulosclerosis. The demographic, clinical and laboratory data are presented in Table 1 and Table 2.

#### 3.1.2. GP Cohort

The GP patients were aged from 24 to 92 years old (median 47 years), 57.0% were females and 43.0% males. The majority were people with secondary (48.8%), followed by higher (24.1%) and primary (16.9%) education. The smallest group was people with a vocational education (10.2%). Their demographic data is presented in Table 3. Clinical data were unavailable.

#### 3.1.3. Basic Comparison of Study Cohorts

The study cohorts were compared considering the available demographic data, comparison is presented in Table 4.

### 3.2. Comparison between CKD and GP Cohort

**Figure 1:**
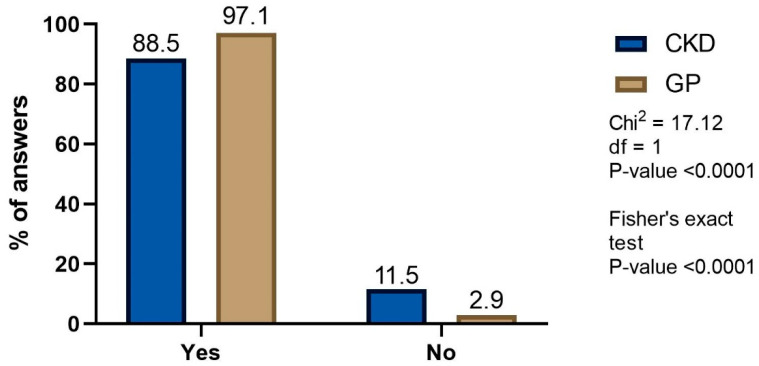
Proportion of CKD and GP patients using OTC NSAIDs or analgesics.

As many as 88.5% of the CKD and 97.1% of GP patients used NSAIDs and/or analgesics and the difference between the study groups was significant (*p* < 0.0001; Figure 1). 

**Figure 2:**
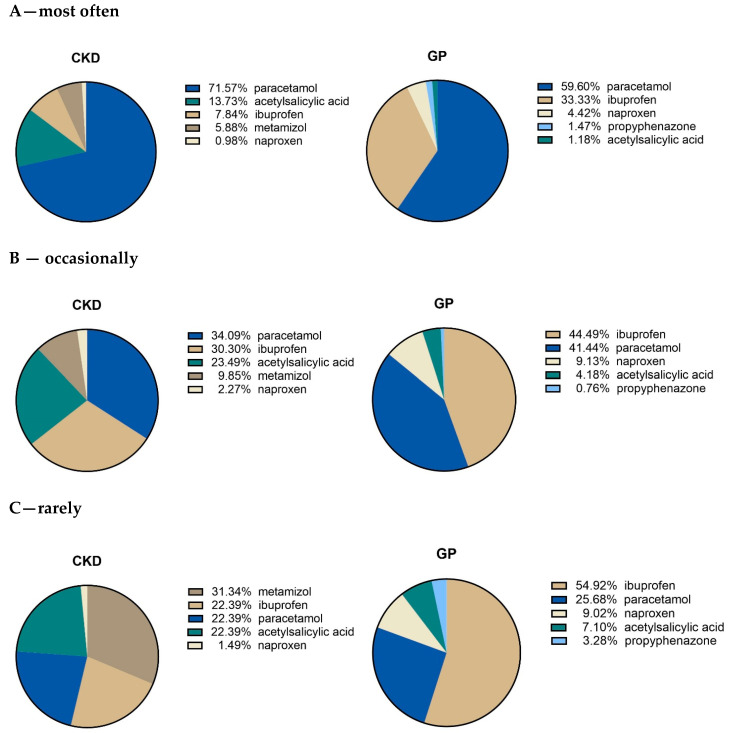
Comparison of the most frequently used NSAIDs and analgesics in CKD and GP patients, based on the Patients’ answers to question 3 (which of the listed drugs are you taking?/please provide the names of analgetic drugs you used–from the most to the least frequently used).

We decided to divide the study participants into the three subgroups, regarding their OTC NSAIDs and/or analgesics frequency of use (A—most often, B—occasionally, C—rarely). Paracetamol was chosen the most often by patients from both study groups, but the proportion of patients taking paracetamol was significantly higher in CKD group (*p* < 0.006; Figure 2A). Interestingly, we found that the less frequently the NSAIDs or analgesics were taken (Figure 2B, Figure 2C vs. Figure 2A), the lower the proportion of paracetamol (34.09%, 31.34% vs. 71.57% for CKD and 41.44%, 25.68 vs. 59.60% for GP) and the higher the proportion of ibuprofen (30.30%, 22.39% vs. 7.84% for CKD and 44.49%, 54,92% and 33.33% for GP) was reported. This indicated to us that CKD patients who reach for these drugs less frequently prefer ibuprofen, acetylsalicylic acid or metamizol, whereas GP patients use mostly ibuprofen. Accordingly, the proportion of patients taking ibuprofen was significantly higher in GP group in all 3 categories of the frequency of the drug use (*p* < 0.0001; *p* = 0.006; *p* < 0.0001; Figure 2A–C). The statistical calculations are presented in Table 5.

**Figure 3:**
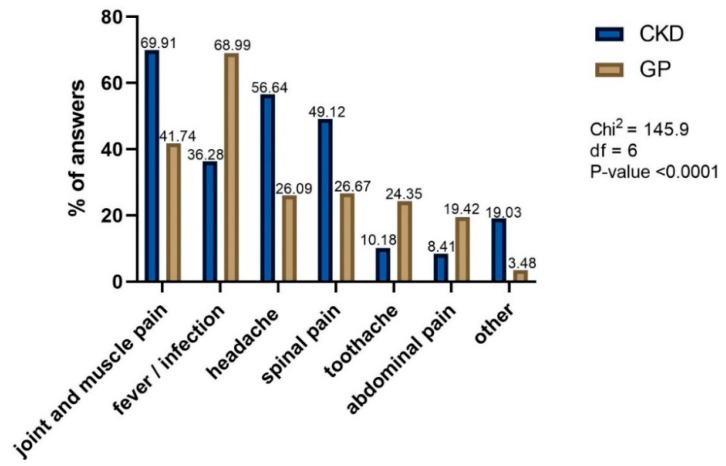
The indications to take OTC NSAIDs or analgesics.

Such significant differences in ibuprofen use between CKD and GP patients may be partially explained by the differences in indications for therapy. The most popular cause of NSAIDs or analgesics use in CKD patients was joint and muscle pain, which was reported almost twice as often as an indication for use in CKD compared to GP patients. Similarly, spinal pain was significantly more often the reason to take these drugs in CKD than in GP patients. In contrast, infection or fever was a more common indications for use of NSAIDs or analgesics in GP patients, in whom they were significantly more often indicated than in CKD patients. Toothache was an indication for NSAIDs and/or analgesics use in twice as many GP patients compared to the CKD group (Figure 3).

Patient gender was found to be associated with acetylsalicylic acid use in CKD patients only. Most probably because of the higher frequency of cardiovascular indications in the CKD group. Other drug use was not linked to gender (Table 6).

**Figure 4:**
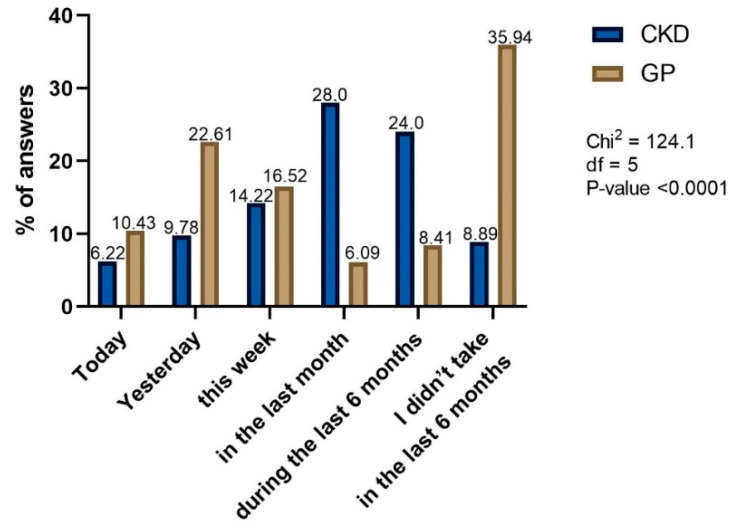
The time from the last dose of OTC NSAIDs or analgesics use.

We also found a significantly higher frequency of NSAIDs or analgesics use in GP compared to CKD patients (Figure 4). As many as 33% of the GP group took NSAIDs or analgesics on the day or the day preceding the survey. In contrast, 28% and 24% of CKD group took these drugs a month or 6 months prior to survey, respectively. 

**Figure 5:**
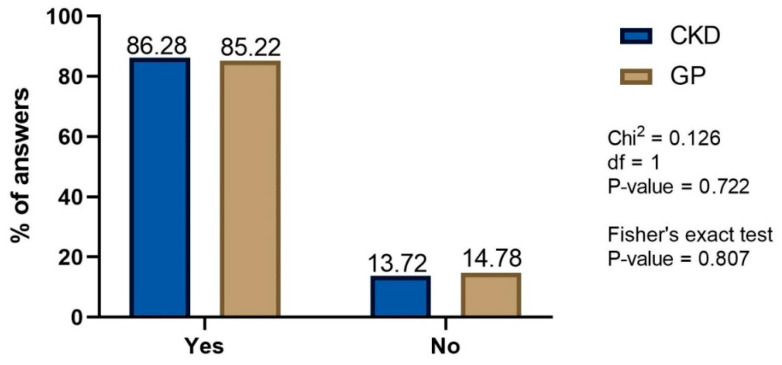
The proportion of patients aware of potential side effects of NSAIDs or analgesics.

No significant differences (Chi^2^ = 0.69, *p*-value = 0.4) were found between the number of CKD and GP patients who read the drug information in the leaflet. Importantly, in both study groups as many as one fourth of patients does not read it at all (26.6 and 23.5% for CKD and GP group, respectively). The awareness of the potential side effects of NSAIDs or painkillers was not significantly different in CKD and GP patients (Figure 5). It is worth to mentioning that the patients’ awareness of the potential side effects of NSAIDs or analgesics is highly satisfactory. Interestingly, as many as 84% of GP respondents stated that they had never observed side effects after taking these drugs.

**Figure 6:**
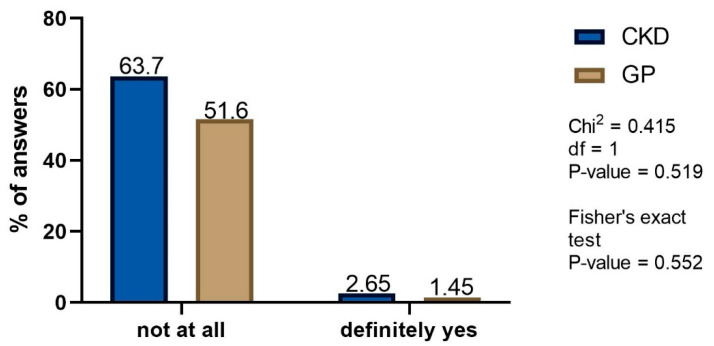
The influence of advertisements on the decision to take NSAIDs or analgesics: CKD—frequency of advertisement—driven decision making; GP—grading of the belief that advertisement is important (merged questions). Only definitive answers are presented.

The influence of advertisements on the decision to take NSAIDs or analgesics was found to be marginal in both studied groups. As many as 63.7% of CKD patients stated that their decision to choose NSAIDs or analgesics was never advertisement-driven (Figure 6). Moreover, 51.6% of GP patients graded their belief that advertisement is important decision factor as very weak (Figure 6).

**Figure 7:**
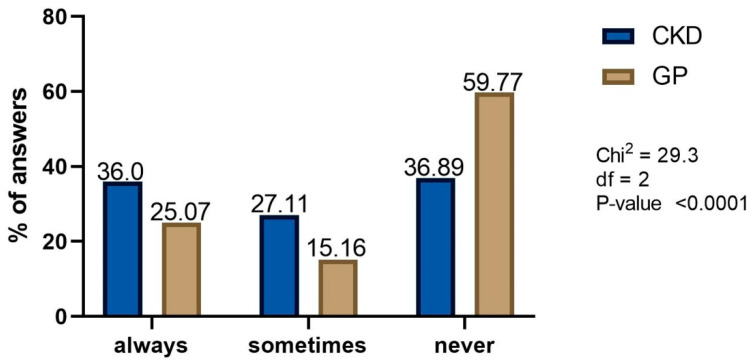
Frequency of medical advice before taking NSAIDs or analgesics.

Almost 60% of GP patients and 37% of CKD patients never go to see a doctor to consult taking NSAIDs or analgesics (Figure 7). Only this difference between the groups is statistically significant.

## 4. Discussion

The results of our study confirmed that NSAIDs and/or analgesics use is very common both in the nephrological and the primary care patient populations: 89 and 97% of patients, respectively. These very high proportions in combination with the age ranges of our populations suggest that patients using OTC painkillers are people of all ages. The elderly were previously reported to be particularly prone to take these drugs for multiple complaints. They are also at higher risk of adverse events [19]. However, young individuals were also reported to use NSAIDs and/or analgesics excessively. In a British study, over two thirds of the university student population took these drugs, and one sixth of them exceeded the maximum dose [2]. 

Interestingly, patients visiting their GP used NSAIDs and analgesics significantly more often than CKD patients. The differences in analgesics and antipyretics consumption between different patient populations are known from the literature. For example, according to the Swedish registry, 34% of elderly people with intellectual disabilities use these drugs, in comparison to 44% of the general population [20]. We previously reported that 64% of liver [4] and 63% of kidney transplant recipients use NSAIDs and/or analgesics [4,5]. In contrast, analgesics and antipyretics were recorded only in 6.6% of patients hospitalized in the internal medicine wards [21]. Such cohort-related discrepancies may be explained by the differences in the indications, population-specific comorbidities, pain burden and medical advice, age, drug education, patients’ beliefs and study methodologies. 

We found paracetamol to be the most frequently used drug in both study groups. However, the proportion of ibuprofen use, the most popular NSAID, was significantly higher in GP than in CKD patients. This observation is in line with previous reports. People with intellectual disabilities were more likely than those in the general population cohort to be prescribed paracetamol for all investigated types of pain, and were less likely to have a prescription for NSAIDs [20]. Paracetamol was also the most frequent among analgesics and antipyretics recorded in patients hospitalized in the internal medicine ward [21]. We hypothesize, that CKD patients pay more attention to their kidney function and are better educated about NSAIDs nephrotoxicity. In fact, the awareness of the adverse events risk associated with NSAIDs and/or analgesics use seems relatively high in our study (86% of CKD respondents). Nevertheless, almost 60% of GP patients and 37% of CKD patients never go to see a doctor to consult taking NSAIDs or analgesics. The better education of CKD patients could explain such difference; they probably know that paracetamol is less harmful than NSAID for their kidneys and make a self-decision to take OTC medications instead of waiting for the consultation. However, previous reports suggests that such analgesics self-use dependence on the type of studied cohort [22]. This is puzzling as it is known that 88% of patients consider doctors to be the most reliable source of information about these drugs [4]. Moreover, in one of the surveys as many as 30% of the respondents reported at least one case of NSAIDs or analgesics misuse [23]. Perhaps another explanation for the high self-decision rate is the fact that only 16% of our GP respondents experienced side effects and thus have a sense of drug safety. According to the available data, the frequency of side effects varies depending on the damaged organ and may be as high as 70% in the case of gastrointestinal diseases [9,10]. However, it should be taken into the account that patients may not experience all of the NSAIDs and/or analgesics side effects. It seems that their asymptomatic course was responsible for the low percentage of reported side effects in our study.

Another very important observation is the fact that advertising had little influence on the drug choice. It might seem that it is their availability and excessive advertisement builds the common belief that their use is safe. Meanwhile, according to our survey, the most important factors motivating the purchase of a particular drug were the previous experience with the drug, dosing method, and to a lesser extent the price of the drug, but not its advertising. These results seem to be an important voice in the discussion of the advantage of patient education over advertising bans in order to reduce the related complications and their consequences.

Our study has some limitations. Firstly, the number of completed questionnaires is not equal in CKD and GP groups for the reason we explained previously. Secondly, the GP group was younger and that might influence the reasons, the frequencies and the type of the OTC taken by both groups. Unfortunately, the surveys in each group were performed in different time points and the questions were in part non-identical. However, the statistical analysis was based on the identical questions only and remained unbiased. Finally, the clinical data from GP group were unavailable and many of the CKD patients are currently lost to follow-up. So, we cannot fully estimate the risk factors of NSAIDs intake entirely.

## 5. Conclusions

As many as 89% of CKD and 97% of GP patients of all ages use NSAIDs and/or analgesics.

Almost 37% of CKD and 60% of GP patients (!) never go to a doctor to consult taking NSAIDs or analgesics

The awareness of the potential side effects of NSAIDs or painkillers is satisfactory (more than 85% in both groups).

The most popular reason of NSAIDs or analgesics use differ between cohorts. Almost 70% of CKD pointed joint and muscle pain, whereas 69% of GP chose infection or fever as a main reason.

Previous patient experience rather than drug advertising drives the decision on NSAIDs and/or analgesics choice, implying educational approaches.

## Figures and Tables

**Table 1:** Demographic, clinical and laboratory characteristics of the CKD group.

**Demographic Data**		
Age	mean (SD)	60.5 (16.0)
	range	19.0–91
	median (IQR)	63.0 (50–72)
Gender, n (%)	Female	141 (62.4)
	Male	85 (37.6)
BMI	mean (SD)	28.0 (4.7)
	range	17.1–39.9
	median (IQR)	63 (24.9–31.1)
Employment status, n (%)	Active	80 (35.4)
	Unemployed or retired	146 (64.6)
**Clinical Data**		
Disease etiology, n (%)	Unknown etiology	65 (30.7)
	IgA nephropathy/HSP Nephritis	24 (10.6)
	Diabetic kidney disease	22 (9.7)
	Focal segmental glomerulosclerosis	21 (9.3)
	Polycystic kidney disease	21 (9.3)
	Nephrectomy	17 (7.5)
	Systemic lupus erythematosus	14 (6.2)
	Urinary tract infections	14 (6.2)
	Membranous nephropathy	9 (3.9)
	Urolithiasis	8 (3.5)
	Membranoproliferative glomerulonephritis	6 (2.6)
	Other glomerulonephritis	5 (2.2)
Comorbidities, n (%)	Diabetes Mellitus	67 (29.6)
	Hypertension	180 (79.6)
	Other	221 (97.8)
**Laboratory Results**		
Serum creatinine (mg/dL)	mean (SD)	1.45 (0.9)
eGFR (mL/min/1.73 m^2^)	mean (SD)	58.16 (27.9)
Protein (g/24 h urine sample, n = 108)	mean (SD)	0.82 (1.17)
Hemoglobin (mg/dL)	mean (SD)	14.17 (9.53)
Platelets (L/L)	mean (SD)	244.8 (70.8)
Albumin (g/dL), serum	mean (SD)	5.4 (4.4)

n—number of patients, BMI—body mass index, IQR—interquartile range, SD—standard deviation, eGFR—estimated glomerular filtration rate, HSP—Henoch-Schoenlein Purpura

**Table 2:** Kidney function in CKD group prior to the survey.

	Years before the Survey				
	−5	−3	−1	Delta (Difference)	*p*-Value
Serum creatinine, (mg/dL)	1.35 (0.49)	1.42 (0.70)	1.47 (0.71)	+0.12	0.35
eGFR, (mL/min/1.73 m^2^*)*	60.6 (27.1)	57.6 (27.5)	54.4 (25.3)	−6.2	0.21

values are set as mean (SD), *p* value is set as <0.05 and calculated with Kruskal-Wallis Test

**Table 3:** Demographic characteristics of the GP group.

Demographic and Clinical Data		
Age	mean (SD)	49 (14.8)
	range	24–92
	median (IQR)	47.00 (35–61)
Gender, n (%)	Female	197 (57.0)
	Male	148 (43.0)
Comorbidities, n (%)	Diabetes Mellitus	43 (12.5)
	Hypertension	122 (35.4)
	Other	67 (19.4)
Residence, n (%)	Village	70 (20.3)
	Town < 100 k inhabitants	62 (18.0)
	Town >100 k inhabitants	212 (61.6)
Education, n (%)	High school	168 (48.8)
	Bachelor’s or Master’s degree	83 (24.1)
	Primary (8th grade)	58 (16.9)
	Vocational (10th grade)	35 (10.2)

n—number of patients, IQR—interquartile range, SD—standard deviation

**Table 4:** Demographic comparison of CKD and GP group.

Demographic and Clinical Data		CKD	GP	*p*-Value
Age	mean (SD)	60.5 (16.0)	49 (14.8)	<0.0001 ^a^
	range	19.0–91	24–92	
	median (IQR)	63.0 (50–72)	47.00 (35–61)	
Gender, n (%)	Female	141 (62.4)	197 (57.0)	0.2986 ^b^
	Male	85 (37.6)	148 (43.0)	
Comorbidities, n (%)	Diabetes Mellitus	67 (29.6)	43 (12.5)	<0.0001 ^b^
	Hypertension	180 (79.6)	122 (35.4)	<0.0001 ^b^

n—number of patients, IQR—interquartile range, SD—standard deviation, ^a^ Mann-Whitney U-Test, ^b^ Fisher’s exact test.

**Table 5:** Statistical evaluation of the most frequently used NSAIDs and analgesics in CKD and GP patients.

Medication	Answer’s Statistics	Frequency					
		A		B		C	
		Group					
		GP	CKD	GP	CKD	GP	CKD
paracetamol	Yes, n (%)	202 (59.6)	146 (71.57)	109 (41.44)	45 (34.09)	94 (25.68)	15 (22.39)
	*p*-value	0.0048 ^a^		0.1575 ^a^		0.5678 ^a^	
		0.0055 ^b^		0.1893 ^b^		0.6474 ^b^	
ibuprofen	Yes, n (%)	113 (33.33)	16 (7.84)	117 (44.49)	40 (30.30)	201 (54.92)	15 (22.39)
	*p*-value	<0.0001 ^a^		0.0066 ^a^		<0.0001 ^a^	
		<0.0001 ^b^		0.0066 ^b^		<0.0001 ^b^	
acetylsalicylic acid	Yes, n (%)	4 (1.18)	28 (13.73)	11 (4.18)	31 (23.49)	26 (7.1)	15 (22.39)
	*p*-value	<0.0001 ^a^		<0.0001 ^a^		<0.0001 ^a^	
		<0.0001 ^b^		<0.0001 ^b^		0.0004 ^b^	
naproxen	Yes, n (%)	15 (4.42)	2 (0.98)	24 (9.13)	3 (2.27)	33 (9.02)	1 (1.49)
	*p*-value	0.0256 ^a^		0.0109 ^a^		0.0353 ^a^	
		0.0384 ^b^		0.0104 ^b^		0.044 ^b^	

^a^—Chi^2^ Test, ^b^—Fisher’s exact Test, n—number of answers, list of selected medications is summarized in Appendix C, A—most often, B—occasionally, C—rarely.

**Table 6:** The association of gender with type of the most frequently used NSAIDs or analgesics.

Medication	CKD				*p*-Value	GP				*p*-Value
	Female		Male			Female		Male		
	n (%)					n (%)				
paracetamol	99	(75.00)	47	(65.28)	0.141 ^a^	113	(57.36)	94	(63.51)	0.248 ^a^
					0.148 ^b^					0.268 ^b^
acetylsalicylic acid	10	(7.58)	18	(25.00)	0.0005 ^a^	3	(1.52)	1	(0.68)	0.467 ^a^
					0.001 ^b^					0.638 ^b^
ibuprofen	10	(7.58)	6	(8.33)	0.847 ^a^	70	(35.53)	43	(29.05)	0.204 ^a^
					0.999 ^b^					0.246 ^b^
metamizol	11	(8.33)	1	(1.39)	0.044 ^a^	0	(0.00)	0	(0.00)	n.a.
					0.059 ^b^					
naproxen	2	(1.52)	0	(0.00)	n.a.	9	(4.57)	6	(4.05)	0.817 ^a^
										0.999 ^b^
propyphenazone	0	(0.00)	0	(0.00)	n.a.	2	(1.02)	4	(2.70)	n.a.

^a^—Chi^2^ Test, ^b^—Fisher’s exact Test, n—number of answers, list of selected medications is summarized in Appendix C.

## Data Availability

Data available upon request.

## Appendix A. The Questionnaire about OTC NSAIDs and/or Analgesics Use in CKD Patients

1. Do you use drugs for pain relief?

(Mark one answer, please)

a. yes

b. no

2. Are they over-the-counter drugs?

(Mark one answer, please)

a. yes

b. no

3. Which of the listed drugs are you taking?

(Please indicate from the most used)

a. .......

b. .......

c. ........

4. Have you taken any other painkillers

(Mark one answer, please)

a. yes

b. no

5. What was the reason you changed your pain medication?

(Mark one answer, please)

a. the drug I was taken finished

b. the doctor recommended a drug change

c. a friend and/or family offered me a drug

e. other............which?

6. Do you carry the pain medication with you?

a. yes

b. no

7. When did you take over-the counter painkiller for the last time?

(Mark one answer, please)

a. today

b. yesterday

c. this week

d. this month

e. during the last 6 months

f. I didn’t take analgesic drugs in the last 6 months

g. hard to say, I don’t remember

8. Do you read the leaflets attached to the over-the counter painkillers before use?

(Mark one answer, please)

a. yes, always

b. yes, sometimes

c. occasionally

d. no, never

9. How much on average do you spend on drugs per month?

(Mark one answer, please)

a. less than 10 PLN

b. 10–50 PLN

c. 50–100 PLN

d. hard to say, I don’t remember

10. You take them because of:

(Select all appropriate variants of the answer, please)

a. toothache

b. headache

c. joint pain

d. menstrual pain

e. back pain

f. other...................?

11. How frequently do you suffer from pain?

(Mark one answer, please)

a. every day

b. several times a week

c. several times a month

d. once in a month

e. few times in a year

f. once a year

g. less often than once a year

h. never

i. hard to say

12. What ways of dealing with pain do you know?

(Select all appropriate variants of the answer, please)

a. painkillers

b. physiotherapy

c. acupuncture

d. massage

e. psychotherapy

f. herbal medicine

g. other.....................

13. Which ways of dealing with pain do you use and how often?

(Select all appropriate variants of the answer, please)

	**Every Day**	**Few Times in a Week**	**Few Times in a Month**	**Once in a Month**	**Few Times in a Year**	**Once in a Year**	**Less Often than Once in a Year**	**Never**	**Hard to Say**
painkillers									
physiotherapy									
acupuncture									
massage									
psychotherapy									
herbal medicine									
other									

14. Do you think that the painkillers may cause side effects?

(Mark one answer, please)

a. definitely yes

b. rather yes

c. sometimes

d. rather no (go to the question 16, please)

e. certainly not (go to the question 16, please)

f. hard to say, I don’t know (go to the question 16, please)

15. What side effects can occur after taking pain medicine?

(Select all appropriate variants of the answer, please)

a. heartburn

b. headache

c. arousal

d. somnolence

e. abdominal pain

f. nausea

g. vomiting

h. diarrhea

i. anemia

j. ulcers in the stomach and/or duodenum

k. gastrointestinal bleeding

l. blood clotting disorders

m. liver damage

n. kidney damage

o. bone marrow damage

p. allergic skin reactions such as: rash, asthma

q. other....................................

16. Do you pay attention to what ingredients are contained in painkillers?

(Mark one answer, please)

a. yes, always

b. yes, sometimes

c. occasionally

d. no, never

17. Do you think multi-component drugs are more effective?

(A multi-component drug combines several painkillers, often with the addition of, for example, caffeine: Saridon, Coffepirine. Fights several symptoms/ailments, e.g., headache, throat pain, exhausting runny nose, cough, etc.)

a. yes

b. no

18. Do you use multi-component drugs?

(Mark one answer, please)

a. yes

b. no

19. Do you consult the choice of painkiller with your doctor?

(Mark one answer, please)

a. yes, always

b. yes, often

c. occasionally

d. no, never

20. Do you think a doctor is a good source of knowledge about pain medicine?

(Mark one answer, please)

a. yes, always

b. yes, often

c. sometimes

d. no, never

e. hard to say

21. From where do you get the information about a given drug most often?

(Mark one answer, please)

a. advertisement

b. internet

c. doctor

d. family

e. friends

f. own experience

22. Do you think that advertisement of painkillers reliably informs you about the effectiveness of the drug?

(Mark one answer, please)

a. yes, always

b. yes, often

c. sometimes

d. no, never

e. hard to say

23. Was your decision to buy an analgesic drug influenced by the message contained in the advertisement?

(Mark one answer, please)

a. yes

b. no

24. Does the dosage of the painkiller matter to you when deciding to use this medicine?

a. has no matter

b. does not matter

c. it has a lot of importance

25. Does the price of the painkillers matter to you when choosing it?

(Mark one answer, please)

a. it has a lot of importance

b. it is of great importance, but is only of the determining factors

c. does not matter

d. has no meaning

e. hard to say

**Metrics**

1. Date of birth (day-month-year)......../......../........

2. Gender F □ M □

3. Do you work professionally?

a. yes

b. no

4. Do you use:

a. vitamins

b. dietary supplements

c. no, I do not use vitamins nor dietary supplements

5. What diseases do you have?

(Select all appropriate variants of the answer, please)

a. hypertension

b. diabetes mellitus

c. gastric ulcers disease and/or duodenal ulcer

d. cardiovascular diseases (ischemic heart disease, myocardial infarction)

e. kidney failure

f. other...........................

6. Are you post-transplant?

(Enter the date, please)

a. kidney (day/month/year)..........

b. liver (day/month/year)..........

c. I’m not post-transplant

## Appendix B. The Questionnaire about OTC NSAIDs and/or Analgesics Use in GP Population

1. Do you use drugs sold over-the-counter for pain relief?

a. yes

b. no

2. How frequently do you suffer from pain?

a. sporadically: several times a year

b. rarely: 1–2 times a month

c. commonly: several times a month

d. often: several times a week

e. very often: every day or several times a day

3. Please provide the names of analgetic drugs you used?

(from the most to the least frequently used)

a............... b................. c................d....................

4. Where do you buy them? (you can select more than one answer)

a. pharmacy

b. kiosk

c. gas station

d. internet

e. supermarket

f. herbal shop

g. other............?

5. You take them because of: (you may select more than one answer)

a. abdominal pain

b. back pain

c. joint pain

d. toothache

e. fever

f. to improve the well-being

g. other...................?

6. What is the dose you take?

a. few tablets a year

b. 0–5 tablets a month

c. 0–5 tablets a week

d. 1–2 tablets a day or about 15 tablets a month

e. more than 3 tablets a day

7. Do you manage pain with analgesic drugs only?

a. yes

b. no

8. If not, what other methods do you use?

a. acupuncture

b. physical therapy

c. massage

d. psychotherapy

e. sport

f. herbal products

g. other..................?

9. Do you read the leaflets attached to the over-the counter painkillers before use?

a. yes

b. no

10. Do you think that the painkillers may cause side effects?

a. yes

b. no

11. If yes, what side effects are you aware of?

(you may indicate more than one answer)

a. diarrhea	g. nausea
b. abdominal pain	h. kidney damage
c. headache	i. bone marrow damage
d. peptic ulcer	j. liver damage
e. allergic skin reactions such as: rash, asthma	k. blood clotting disorders
f. arousal or somnolence	

12. Have you ever experienced side effects from using the over-the counter painkillers?

a. yes

b. no

13. Do you consult the choice of painkiller with your doctor?

a. always

b. sometimes

c. never

14. How strong do you believe in different sources of information about analgesic drugs?

Please grade your answers from 1 to 5, where:

1. I do not believe at all

2. I believe a little

3. I believe quite a lot

4. I believe very much

5. I believe totally

doctor	I don’t believe at all 1---2---3---4---5 I believe totally
advertisement/media	I don’t believe at all 1---2---3---4---5 I believe totally
family/relatives	I don’t believe at all 1---2---3---4---5 I believe totally
internet	I don’t believe at all 1---2---3---4---5 I believe totally

15. What is important for you about analgesic drugs?

Please grade your answers from 1 to 5, where:

1. unimportant

2. important a little

3. important quite a lot

4. important a lot

5. very important

dosing	unimportant 1---2---3---4---5 very important
price	unimportant 1---2---3---4---5 very important
previous experience	unimportant 1---2---3---4---5 very important
advertised drug efficacy	unimportant 1---2---3---4---5 very important

16. Which of the following diseases do you suffer from?

(you may indicate more than one answer)

a. hypertension

b. diabetes

c. gastric and/or duodenal ulcer disease

d. cardiovascular diseases (coronary disease, myocardial infarction)

e. chronic renal disease

f. chronic liver disease

g. other.......................................?

17. When was the last time you took over-the-counter painkiller?

a. today

b. yesterday

c. this week

d. in the last month

e. during the last 6 months

f. I didn’t take analgesic drugs in the last 6 months

18. In your opinion, the amount of analgesic drugs you take is:

a. small

b. large

19. Please, indicate your gender: F □ M □

20. Please, provide your birth date (day/month/year)...../......./...........

21. What is your place of residence?

a. city over 100,000 inhabitants

b. city below 100,000 inhabitants

c. village

22. What is your education level?

a. basic education

b. secondary education

c. higher education

d. other........................?

## Appendix C. The List of OTC NSAIDs and Analgesics Included in the Questionnaire Supplement

**Main Active Substance**	**Pharmaceutical Formulation** **(Dose)**	**Product Name** **(in Polish)**	**Product ID**
acetylsalicylic acid	acidum acetylsalicylicum (300 mg); ethenezamidum (100 mg); coffeinum (50 mg)	*Etopiryna*	11
	acidum acetylsalicylicum (400 mg); coffeinum (50 mg)	*Kopiryna*	18
	acidum acetylsalicylicum (450 mg); coffeinum (50 mg)	*Coffepirine*	8
	acidum acetylsalicylicum (500 mg)	*Aspirin*	6
	acidum acetylsalicylicum (500 mg); glycinum (200 mg)	*Asprocol*	7
ibuprofen	dexibuprofenum (200 mg)	*Seractil*	29
	ibuprofenum (200 mg)	*Ibupar*	13
	ibuprofenum (200 mg)	*Ibuprofen*	14
	ibuprofenum (200 mg)	*Ibum*	15
	ibuprofenum (200 mg)	*Ibuprom Sprint Caps*	16
	ibuprofenum (200 mg); codeini pfosphas (128 mg)	*Nurofen Plus*	23
	ibuprofenum (200 mg); pseudoephedrini hydrochloridum (30 mg)	*Modafen*	19
	ibuprofenum (400 mg)	*Ibuprom Max*	17
	ibuprofenum (400 mg)	*Nurofen Ultra Forte*	22
metamizol	metamizolum natricum (500 mg)	*Pyralginum*	26
naproxen	naproxenum (200 mg)	*Naproxen*	20
	naproxenum (200 mg)	*Natrax 200*	21
	naproxenum natricum (200 mg)	*Aleve*	2
paracetamol	paracetamolum (250 mg); propyphenazonum (150 mg); coffeinum (50 mg)	*Saridon*	28
	paracetamolum (300 mg)	*Acenol*	1
	paracetamolum (500 mg)	*Apap*	5
	paracetamolum (500 mg)	*Codipar*	9
	paracetamolum (500 mg)	*Efferalgan*	10
	paracetamolum (500 mg)	*Panadol*	25
	paracetamolum (500 mg)	*Paracetamol*	27
	paracetamolum (500 mg); codeini phosphas (15 mg)	*Antidol 15*	3
	paracetamolum (500 mg); coffeinum (30 mg); kodeine (8 mg)	*Solpadeine*	30
	paracetamolum (500 mg); coffeinum (65 mg)	*Apap EXTRA*	4
propyhenazone	propyphenazonum (200 mg); noramidopyrine methanosulfones (300 mg)	*Gardan P*	12
	propyphenazonum (220 mg); allobarbitalum (220 mg)	*Pabialgin P*	24

## Appendix D. Merged Questionnaires

**Figure**	**CKD—Questionnaire**		**GP—Questionnaire**	
	**Question**	**Answer**	**Question**	**Answer**
Figure 1	Do you use drugs for pain relief?	a. yes b. no	Do you use drugs sold over-the-counter for pain relief?	a. yes b. no
Figure 2	Which of the listed drugs are you taking? (Please indicate from the most used)	a…. b… c…	Please provide the names of analgetic drugs you used?(from the most to the least frequently used)	a…. b… c… d… * c and d were merged in calculations
Figure 3	You take them (painkillers) because of: (Select all appropriate variants of the answer, please) *	a. toothache c. headache d. joint pain e. menstrual pain f. back pain g. other……………….? * according to patient’s answers in variant ‘other’ we categorized the data as follow: joint and muscle pain; fever/infection; headache; spinal pain; toothache; abdominal pain; other	You take them (painkillers) because of: (you may select more than one answer) *	a. abdominal pain c. back pain d. joint pain e. toothache f. fever g. to improve the well-being h. other…………?
Figure 4	When did you take over-the counter painkiller for the last time? (Mark one answer, please)	a. today b. yesterday c. this week d. this month e. during the last 6 months f. I didn’t take analgesic drugs in the last 6 months g. hard to say, I don’t remember * answer ‘g’ was not included in calculations	When was the last time you took over-the-counter painkiller?	a. today b. yesterday c. this week d. in the last month e. during the last 6 months f. I didn’t take analgesic drugs in the last 6 months
Figure 5	Do you think that the painkillers may cause side effects? (Mark one answer, please)	a. definitely yes b. rather yes c. sometimes d. rather no e. certainly not f. hard to say, I don’t know * a, b, c and d, e, f were merged separately in calculations	Do you think that the painkillers may cause side effects?	a. yes b. no
Figure 6	Do you think that advertisement of painkillers reliably informs you about the effectiveness of the drug? (Mark one answer, please)	a. yes, always b. yes, often c. sometimes d. no, never e. hard to say * only definitive answers were compared	How strong do you believe in different sources of information * (advertisement/media) about analgesic drugs? Please grade your answers from 1 to 5.	1—I do not believe at all 2—I believe a little 3—I believe quite a lot 4—I believe very much 5—I believe totally
Figure 7	Do you consult the choice of painkiller with your doctor? (Mark one answer, please)	a. yes, always b. yes, often c. occasionally d. no, never * a and b were merged in calculations	Do you consult the choice of painkiller with your doctor?	a. always b. sometimes c. never
Table 1, Table 2, Table 3 and Table 4	**Metrics**			
	Gender	a. F b. M	Please, indicate your gender	a. F b. M
	Date of birth (day/month/year)	……..	Please, provide your birth date (day/month/year)	……..
	What diseases do you have? (Select all appropriate variants of the answer, please)	a. hypertension b. diabetes mellitus c. gastric ulcers disease and/or duodenal ulcer d. cardiovascular diseases (ischemic heart disease, myocardial infarction) e. kidney failure f. other ………?	Which of the following diseases do you suffer from? (you may indicate more than one answer)	a. hypertension b. diabetes c. gastric and/or duodenal ulcer disease d. cardiovascular diseases (coronary disease, myocardial infarction) e. chronic renal disease f. chronic liver disease g. other ………?
* do not need any explanation, this is information from author about how the questionnaires were interpreted.				
